# The Impact of ApoE and FOXO3 Genotype on the Risk of Intracerebral Hemorrhage Among American Men of Japanese Ancestry

**DOI:** 10.1093/geroni/igab046.1420

**Published:** 2021-12-17

**Authors:** Kazuma Nakagawa, Randi Chen, Steven Greenberg, G Ross, Bradley Willcox, Kamal Masaki

**Affiliations:** 1 University of Hawaii and Kuakini Medical Center, Honolulu, Hawaii, United States; 2 Kuakini Medical Center, Honolulu, Hawaii, United States; 3 Massachusetts General Hospital, Boston, Massachusetts, United States; 4 VA Pacific Islands Health Care System, Honolulu, Hawaii, United States; 5 Kuakini Medical Center, Kuakini Medical Center/Honolulu, Hawaii, United States

## Abstract

This study assessed the impact of APOE e2, e4 minor alleles and the FOXO3 longevity-associated genotype (carrier of SNP rs2802292 “G” allele) on 34-year incidence of intracerebral hemorrhage (ICH). Cox regression models were performed to assess the impact of the APOE e2, e4 and FOXO3 G alleles on the incidence of ICH. A total of 6483 participants were eligible for the analyses. 213 participants developed ICH. Cox-regression model showed neither APOE minor allele vs. common genotype (APOE e3/e3: RR 0.89, 95% CI: 0.64-1.22, p=0.46) nor FOXO3 G carrier status (RR 0.97, 95% CI: 0.72-1.29, p=0.82) was associated with incident ICH. Conversely, both hypertension (RR: 1.46, 95% CI: 1.07-2.00, p=0.02) and low cholesterol level (RR: 0.99, 95% CI: 0.99-1.00, p=0.001) were associated with incident ICH. Carriage of APOE e2 or E4 alleles and the FOXO3 G allele do not appear to impact risk of ICH over 34 years in this cohort.

